# Inefficiency Rates of Biological Immunosuppressive Induction Agents Used in Organ Transplantation: A Pharmacovigilance Study

**DOI:** 10.3390/jcm14103409

**Published:** 2025-05-13

**Authors:** Anca Butuca, Laurentiu Stoicescu, Mirela Livia Popa, Carmen Maximiliana Dobrea, Adriana Muntean, Claudiu Morgovan, Corina Pienar, Felicia Gabriela Gligor, Steliana Ghibu, Ioana Rada Popa Ilie, Adina Frum

**Affiliations:** 1 Preclinical Department, Faculty of Medicine, “Lucian Blaga” University of Sibiu, 550169 Sibiu, Romania; anca.butuca@ulbsibiu.ro (A.B.); claudiu.morgovan@ulbsibiu.ro (C.M.); felicia.gligor@ulbsibiu.ro (F.G.G.); adina.frum@ulbsibiu.ro (A.F.); 2 Internal Medicine Department, Faculty of Medicine, “Iuliu Haţieganu” University of Medicine and Pharmacy, 400000 Cluj-Napoca, Romania; laurentiu.stoicescu@umfcluj.ro; 3 Cardiology Department, Clinical Municipal Hospital, 400139 Cluj-Napoca, Romania; 4 Clinic Medical Department, Faculty of Medicine, “Lucian Blaga” University of Sibiu, 550169 Sibiu, Romania; 5 Clinical Institute of Urology and Renal Transplant Cluj-Napoca, 4-6 Clinicilor Str., 400006 Cluj-Napoca, Romania; munteana2@yahoo.com; 6 Department of Pediatrics, 2nd Pediatrics Clinic, “Victor Babes” University of Medicine and Pharmacy, 300041 Timisoara, Romania; pienar.corina@umft.ro; 7 Department of Pharmacology, Physiology and Pathophysiology, Faculty of Pharmacy, “Iuliu Hatieganu” University of Medicine and Pharmacy, 400012 Cluj-Napoca, Romania; steliana.ghibu@umfcluj.ro; 8 Department of Endocrinology, Faculty of Medicine, “Iuliu Haţieganu” University of Medicine and Pharmacy, 3-5 Louis Pasteur Street, 400349 Cluj-Napoca, Romania; ioana.ilie@umfcluj.ro

**Keywords:** immunosuppressive induction agents, basiliximab, anti-thymocyte globulin, drug resistance, drug ineffectiveness, transplant rejection

## Abstract

Effective immunosuppressant pharmacotherapy is essential for successful organ transplantation. **Background/Objectives**: Generally, induction therapy includes basiliximab (BAS) or anti-thymocyte globulin (THY). However, other biological molecules have been used to accelerate firm immunosuppression. A reduced effectiveness of these induction agents increases the risk of graft rejection. This study aims to evaluate the ineffectiveness rate of biological molecules based on spontaneous reports uploaded to the EudraVigilance database. **Methods**: Specific topics related to the safety profiles of alemtuzumab, BAS, belatacept, and THY were analyzed. A total of 23 preferred terms describing drug resistance, drug ineffectiveness, and transplant rejection were used as the inclusion criteria. Descriptive and disproportionality analyses were performed. **Results**: Regarding the four molecules, 18,564 safety reports were communicated, with *n* = 5089 (27.4%) for THY and *n* = 3469 (18.7%) for BAS. Most adverse drug reactions (ADRs) for THY, BAS, and belatacept affected the adult male population. As expected, the majority of the ADRs were linked to infections, followed by general disorders. BAS presented higher probabilities of drug resistance and transplant rejection being reported among the four molecules. A higher probability of reporting drug ineffectiveness was noted for THY than for the other molecules. **Conclusions**: All the molecules showed small frequencies regarding resistance. As expected, transplant rejection was more frequently reported for all molecules (especially for BAS), accompanied by a notable variability in reporting frequencies. However, a causal relationship between the reported adverse reactions and drug efficacy cannot be established based on the present results. Further real-world evidence studies will enhance our understanding of the safety and efficacy of these drugs in transplant patients.

## 1. Introduction

Organ transplantation has achieved fulminant success over the last few decades due to a combined approach involving precision matching, surgical techniques, and effective immunosuppressant pharmacotherapy. In 2023, over 170,000 transplants were performed worldwide, 9.5% more than the previous year [1]. Kidney transplants accounted for the majority, making up more than half of the total [1], followed by liver transplants [2] and, to a much lesser extent, pancreas, intestine, and heart transplants [3,4,5].

The life expectancy of organ receivers depends on graft survival [6]. Donor–recipient compatibility is assessed through histocompatibility testing; however, even with high compatibility, lifelong immunosuppressive therapy is still required [7]. The human leukocyte antigen (HLA) system is involved in identifying “self” components and in triggering the immune response against “non-self” elements. It is highly polymorphic, and a good match to the donor increases the chances of organ acceptance, reducing allograft immunogenicity [8].

Regarding pharmacotherapy, international guidelines mention two major stages: induction and maintenance [9,10]. Initial regimen standard recommendations include calcineurin inhibitors, mycophenolate, steroids, and induction therapy using basiliximab (BAS) or anti-thymocyte globulin (THY) [9]. Belatacept [9], alemtuzumab [11], eculizumab [12], and rituximab [10] are other biological molecules that have been successfully used for the same purpose. This group contains molecules that have been newly introduced into the market. Several studies concerning both effectiveness [13,14] and cost-effectiveness have shown the benefits of antibodies for induction therapy [15,16] at an individual level and for health systems. Maintenance therapy is conducted with calcineurin inhibitors, mycophenolate, and steroids. The doses of steroids (prednisolone or methylprednisolone) and calcineurin inhibitors (tacrolimus—elective; cyclosporine—alternative) are reduced in later post-operative stages [9].

The medicinal products recommended for inducing immunosuppression have different mechanisms of action. BAS and rituximab are chimeric monoclonal antibodies. BAS acts on the surface of activated T-lymphocytes, blocking the CD25 antigen; specifically, the alpha chain of interleukin-2 receptors [17]. Rituximab binds to the CD20 antigen of B-cells and is used “off-label” for immunosuppression in transplant patients [18]. Its approved indications are CD20-positive B-cell non-Hodgkin’s lymphoma, chronic lymphocytic leukemia, rheumatoid arthritis, microscopic polyangiitis, granulomatosis with polyangiitis, and pemphigus vulgaris. Off-label uses include myasthenia gravis, multiple sclerosis, chronic steroid-refractory GVHD, Hodgkin lymphoma, systemic lupus erythematosus, thrombotic thrombocytopenic purpura, Waldenstrom macroglobulinemia, and membranous nephropathy [19]. Alemtuzumab and eculizumab are humanized monoclonal antibodies. The former binds to CD52 and exerts a strong immunosuppressive effect by targeting multiple immune cell lines [20], while the latter binds to the complement protein C5 [12]. Belatacept is a protein that inhibits T-cell co-stimulation [21]. An intense immunosuppressive response is triggered by anti-thymocyte globulin, which consists of derived antibodies against T-cells, targeting both mature and immature forms [22] due to the antibodies’ ability to bind to a vast number of targets (e.g., CD1a, CD3/TR, CD4, CD6, CD7, CD8, CD16, CD25, CD30, CD32, CD 80, HLA class I heavy chains, and others) [23,24].

Two critical aspects are the main advantages of immunosuppression induction therapy; these aspects are the time of response and potency, aimed at accelerating firm immunosuppression. A reduced effectiveness of these induction agents increases the risk of graft rejection [15,16].

EudraVigilance (EV) is a platform launched by the European Medicines Agency in 2012, the main objective of which is to collect spontaneous reports of suspected adverse drug reactions (ADRs) [25,26,27,28]. In addition, disproportionality analysis is a validated method used in post-marketing drug safety surveillance.

This study evaluates the ineffectiveness rate of biological molecules used as immunosuppressant inductors based on real-world data comprising spontaneous reports uploaded to the EudraVigilance database.

## 2. Materials and Methods

### 2.1. Study Design

Based on individual case safety reports (ICSRs) uploaded to the EudraVigilance (EV) database up to 19 January 2025, available on https://www.adrreports.eu/, accessed on 22 January 2025, we conducted a pharmacovigilance study on ADRs reported for biological immunosuppressive induction agents used in transplant therapy (BIIAs). Individual case safety reports of four BIIAs (alemtuzumab, BAS, belatacept, and THY) were analyzed. Although many reports were uploaded to EV for eculizumab and rituximab, they were not included in the present study because immunosuppression induction in transplants is not their main indication, which may represent a confounding factor. Using data extracted on 21 January 2025, descriptive and disproportionality analyses were performed.

### 2.2. Descriptive Analysis

The first stage of this study comprises analyzing the distribution of ICSRs reported for BIIAs: alemtuzumab, BAS, belatacept, and THY. Further, a comparison of general characteristics data reported for all BIIAs was performed. Each ICSR contains four data categories: (i) patient age: 0–1 month, 2 months–2 years, 3–11 years, 12–17 years, 18–64 years, 65–85 years, over 85 years, or not specified (NS); (ii) sex (male, female, or NS); (iii) geographic origin of reports (European Economic Area (EEA), non-EEA, or NS); (iv) reporter category (healthcare professional (HP), non-HP, or NS). Subsequently, the frequency of reports with ADRs in each SOC, serious cases, and cases distributed based on SOCs were comparatively analyzed for the four BIIAs. Finally, a comparison between ADRs related to drug resistance, ineffectiveness, and transplant rejection was conducted. According to European Medicines Agency regulations, different preferred terms (PTs) can be used for reporting ADRs. In the present study, 2 PTs were used to evaluate drug resistance, 11 to evaluate drug ineffectiveness [29], and 11 to evaluate transplant rejection (Table 1).

### 2.3. Disproportionality Analysis

To establish the probability of ADR reporting, a disproportionality analysis was conducted. For each ADR group (drug resistance, drug ineffectiveness, and transplant rejection) reported for each BIIA, this probability was evaluated by comparing drugs used in related therapeutic areas or similar clinical contexts. Reporting odds ratios (RORs) and 95% confidence intervals (95% CI) must be calculated to identify similarities and differences in ADR reporting in EV [30,31]. The disproportionality analysis can be validated if a minimum of five cases is reported for each ADR and the lower limit of the 95% CI is above 1 [32,33,34]. The MedCalc application (retrieved from https://www.medcalc.org/calc/odds_ratio.php (Version 23.1.3) accessed on 28 January 2025) was used to calculate RORs and 95% CIs [35].

## 3. Results

### 3.1. Descriptive Analysis

#### 3.1.1. ICSR Analysis

**Figure 1:**
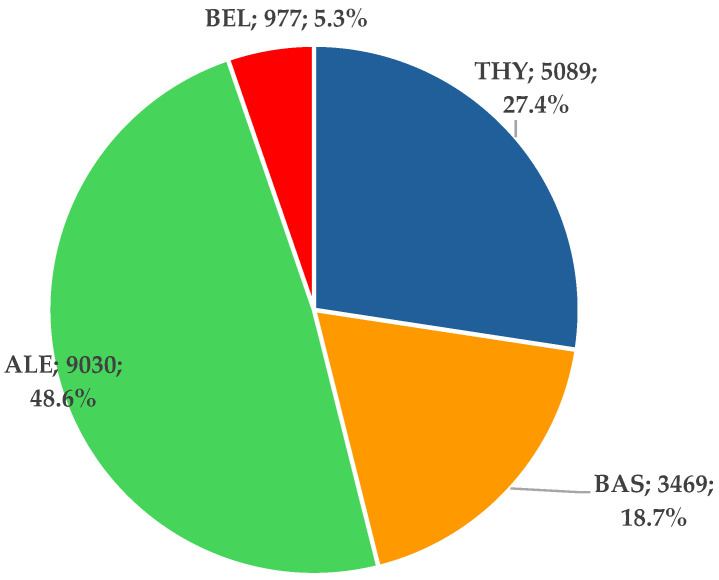
Distribution of ICSRs reported for biological immunosuppressive induction agents used in transplant therapy. ALE—alemtuzumab; BAS—basiliximab; BEL—belatacept; THY—anti-T lymphocyte immunoglobulin for human use (rabbit).

A total of 18,565 ICSRs were reported for the four BIIAs; 48.6% of the total (*n* = 9030) were related to alemtuzumab. The ICSRs reported for THY (*n* = 5089) and BAS (*n* = 3469)—specific biologicals used in post-transplant induction therapy—represented 27.4% and 18.7% of the total, respectively (Figure 1).

According to the data presented in Table 2, the majority of ICSRs related to THY (50.9%), BAS (58.4%), and alemtuzumab (56.7%) are registered in the 18–64-year age group. Additionally, the majority of reports on the use of THY (47.1%), BAS (55.1%), and belatacept (51.2%) were recorded for male patients. Except for alemtuzumab (38.1%), the origin of the reports was non-EEA (THY—67.6%; BAS—73.1%; belatacept—63.2%). Furthermore, HP was the main category of these reports.

#### 3.1.2. ICSR Analysis

**Figure 2:**
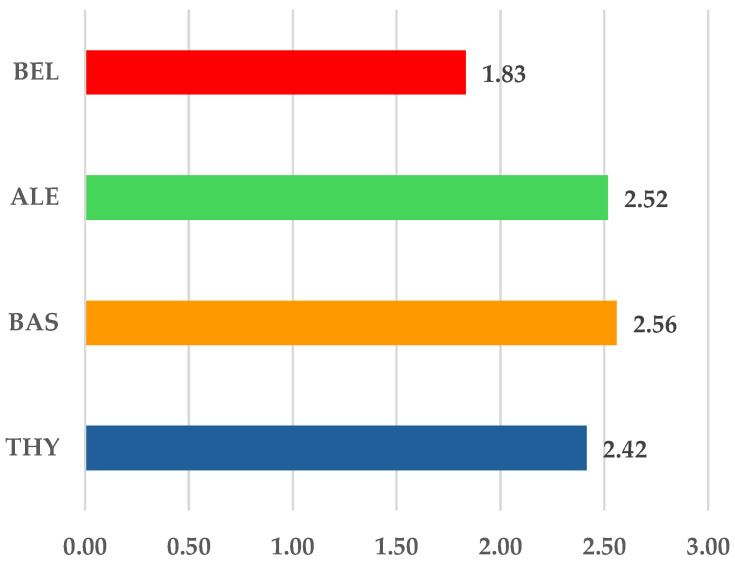
The average number of SOCs reported for each ICSR. ALE—alemtuzumab; BAS—basiliximab; BEL—belatacept; THY—anti-T lymphocyte immunoglobulin for human use (rabbit).

In this step, we calculated the frequency of SOCs per ICSR. The lowest value was calculated for belatacept (1.83) and the highest for BAS (2.56). Except for belatacept, all other BIIAs presented a similar ratio to BAS (Figure 2).

**Figure 3:**
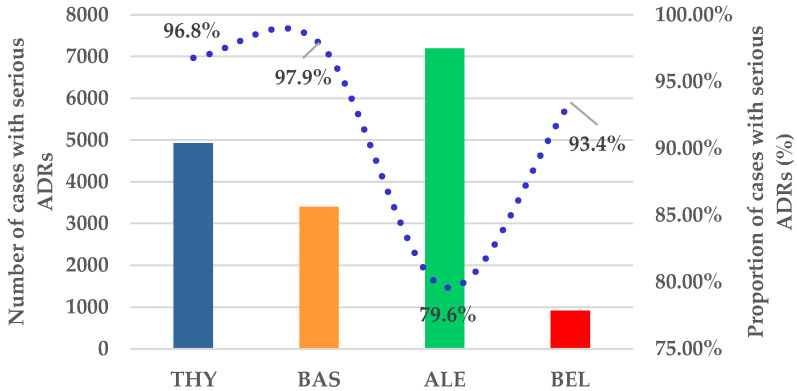
Comparison between cases with serious ADRs reported for BIIAs. ALE—alemtuzumab; BAS—basiliximab; BEL—belatacept; THY—anti-T lymphocyte immunoglobulin for human use (rabbit).

Figure 3 presents the ratio of cases with serious ADRs. Serious conditions were related to all drugs in a high percentage. Thus, over 90% of serious cases were observed for THY, BAS, and belatacept.

The highest relative SOC occurrence frequencies were recorded for the following categories (Table 3): (i) “General disorders and administration site conditions” (THY—11.5%; BAS—8.2%; alemtuzumab—11.9%; belatacept—8.8%); (ii) “Infections and infestations” (THY—15.4%; BAS—20.3%; alemtuzumab—11.1%; belatacept—23.1%); (iii) “Injury, poisoning, and procedural complications” (belatacept—15.0%); (iv) “Immune system disorders” (THY—10.7%; BAS—12.5%), and (v) “Nervous system disorders” (alemtuzumab—8.5%).

##### ADRs Related to Resistance, Ineffectiveness, and Transplant Rejection

**Figure 4:**
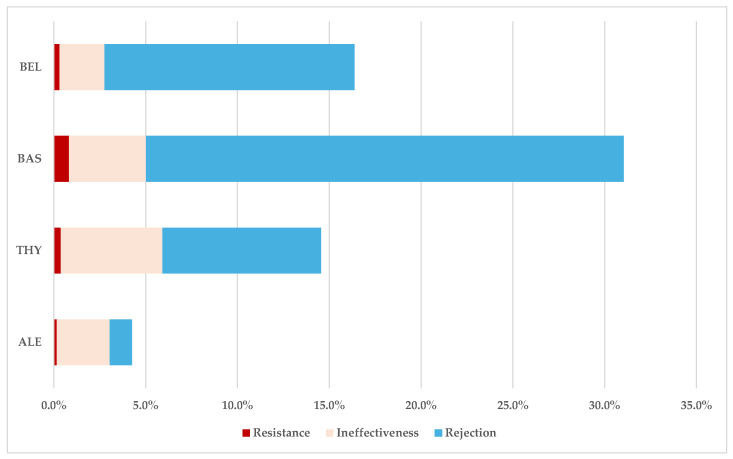
Frequencies of drug resistance, transplant rejection, and drug ineffectiveness in total cases. ALE—alemtuzumab; BAS—basiliximab; BEL—belatacept; THY—anti-T lymphocyte immunoglobulin for human use (rabbit).

According to Figure 4, the highest frequency of total ADRs related to drug resistance, transplant rejection, and drug ineffectiveness in the total cases was registered for BAS (31%), and the lowest frequency was observed for alemtuzumab (4%). Furthermore, alemtuzumab presented a lower proportion of resistance and rejection than the other BIIAs.

### 3.2. Disproportionality Analysis

#### 3.2.1. Drug Resistance

**Figure 5:**
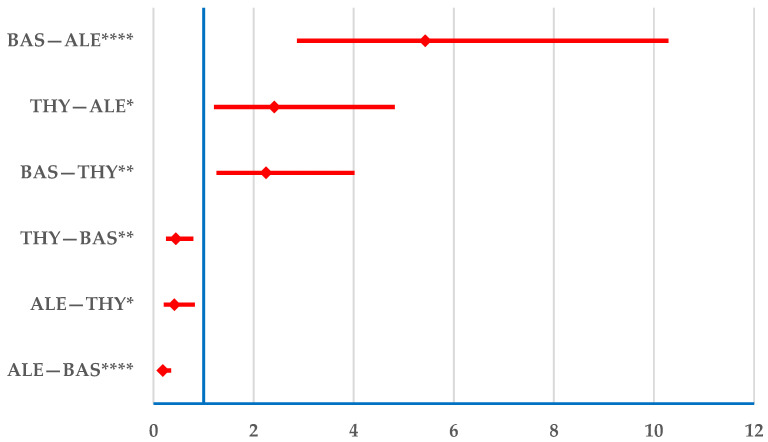
Disproportionality analysis of drug resistance. ALE—alemtuzumab; BAS—basiliximab; THY—anti-T lymphocyte immunoglobulin for human use (rabbit). * *p* < 0.05; ** *p* ≤ 0.01; **** *p* ≤ 0.0001.

Alemtuzumab has a lower probability of reporting resistance than BAS (ROR: 0.2; 95% CI: 0.1–0.3) and THY (ROR: 0.4; 95% CI: 0.2–0.8); conversely, BAS has a higher probability of reporting resistance (Figure 5). In addition, a higher probability of reporting was observed for THY than alemtuzumab. For belatacept, only three signals were documented in this category. As at least five cases are needed to compute the ROR, belatacept was excluded from this analysis (Figure 5).

#### 3.2.2. Drug Ineffectiveness

**Figure 6:**
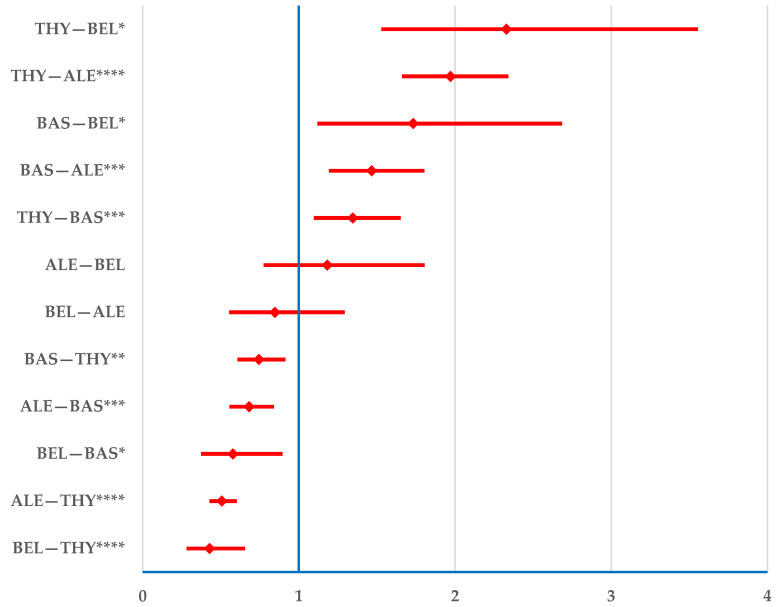
Disproportionality analysis of drug inefficacy. ALE—alemtuzumab; BAS—basiliximab; BEL—belatacept; THY—anti-T lymphocyte immunoglobulin for human use (rabbit). * *p* < 0.05; ** *p* ≤ 0.01; *** *p* ≤ 0.001; **** *p* ≤ 0.0001.

Alemtuzumab and belatacept had a lower probability of reporting drug ineffectiveness than THY (alemtuzumab—ROR: 0.5; 95% CI: 0.4–0.6; belatacept—ROR: 0.4; 95% CI: 0.3–0.7) and BAS (alemtuzumab—ROR: 0.7; 95% CI: 0.6–0.8; belatacept—ROR: 0.6; 95% CI: 0.4–0.9). No difference was observed between belatacept and alemtuzumab (Figure 6).

#### 3.2.3. Transplant Rejection

**Figure 7:**
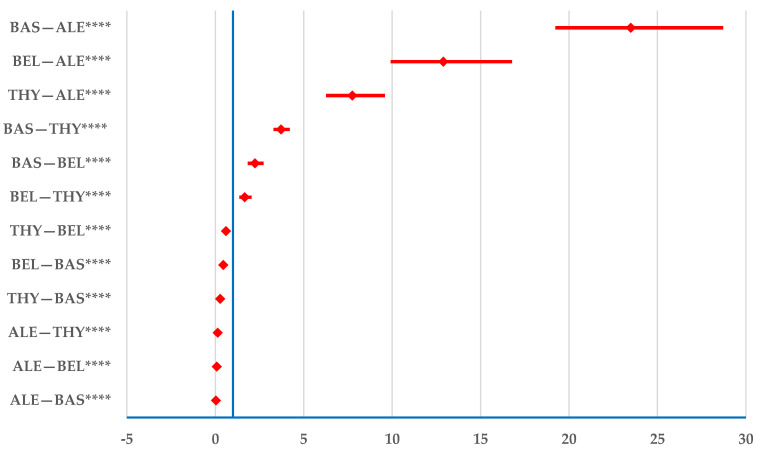
Disproportionality analysis of transplant rejection. ALE—alemtuzumab; BAS—basiliximab; BEL—belatacept; THY—anti-T lymphocyte immunoglobulin for human use (rabbit). **** *p* ≤ 0.0001.

Regarding transplant rejection, alemtuzumab has a lower probability of being reported than all other BIIAs: BAS—ROR: 0.03; 95% CI: 0.03–0.04; THY—ROR: 0.13; 95% CI: 0.10–0.16; and belatacept—ROR: 0.08; 95% CI: 0.06–0.10. A lower probability of reporting rejection was observed for THY than for BAS (ROR: 0.27; 95% CI: 0.24–0.30) and belatacept (ROR: 0.60; 95% CI: 0.49–0.74) (Figure 7).

## 4. Discussion

Antibody induction therapy has increased substantially over the last 20 years [36]. Most reports uploaded to EV for this drug class involve adult subjects. In our study, specific data detailing the prevalence of ADRs for THY, BAS, and alemtuzumab were limited, but other research groups have reported ADRs associated with these drugs.

There were no major differences in gender in our analysis (Table 2), but the highest proportion of ADRs was recorded for men: THY, 47.10%; BAS, 55.1%; and belatacept, 51.2%. Our results agree with a multinational European study showing that fewer women than men undergo kidney transplants or receive induction therapy [37]. Alemtuzumab showed a different situation, presenting most reports (56.7%) for women. Another recent study reported 10 patients who developed fatal adverse effects during alemtuzumab treatment. Among these patients, one was male and nine were female, suggesting a higher incidence of adverse reactions in women in this small group [38].

Regarding the geographical origin of reports, our findings partially align with the results presented by Sheng et al., who identified the highest number of ADR reports coming from India [39], except alemtuzumab, for which the majority were filed from within the EEA.

Our findings show an over 90% frequency of serious ADRs for all BIIAs, except alemtuzumab (Figure 3). These results are supported by multiple studies. A clinical trial found that alemtuzumab therapy may be an alternative to glucocorticoid-resistant, recurrent, or severe acute kidney transplant rejection, with fewer infusion-related adverse events than THY. This research also suggests that alemtuzumab provides a safer profile for transplant recipients, reinforcing the idea that its lower rate of serious adverse reactions is clinically relevant [40].

The finding that the most frequently reported SOCs are “General disorders and administration site conditions”, “Infections and infestations”, and “Injury, poisoning, and procedural complications” is strongly supported by the literature. One investigation found that alemtuzumab induces prolonged lymphocyte depletion, leading to an increased risk of opportunistic infections, and proposed lower doses to reduce this risk [41]. This aligns with the observation that infections are a major concern in alemtuzumab-treated patients and are frequently categorized as “Infections and infestations”. A detailed examination reported infusion-related side effects following THY administration, which were associated with a significant increase in serum cytokine levels, particularly interleukin-6, indicating that THY can induce cytokine release syndrome [42]. All these findings confirm that THY triggers a strong immune response, leading to high-grade fevers, chills, and severe immune system overactivation. This aligns with the classification of adverse events under “Immune system disorders” for THY, reinforcing the hypothesis that a high frequency of adverse reactions for this drug is reported under this category. Aligning with our results categorized by SOC, a clinical trial reported that hypotension was the most common ADR for THY, occurring in 60% of cases, followed by hyperthermia (40%), agitation (30%), tachycardia (20%), hypertension (15%), tremors (10%), and nausea (5%) [43].

For alemtuzumab, similar results concerning infections, nervous disorders, general disorders, and blood disorders were reported by Zaza et al., indicating that alemtuzumab is associated with pyrexia, rigors, nausea, hypotension, urticaria, dyspnea, rash, emesis, and bronchospasm [44]. A study that examined hematologic complications in lung transplant recipients found that patients receiving induction therapy with alemtuzumab or BAS developed bone marrow failure [45]. A research study on more than 1500 adverse drug event reports uploaded to FAERS—another well-established pharmacovigilance database—identified 295 preferred terms across 24 system organ classifications, with increased blood creatinine and pyrexia being the most frequently reported adverse events associated with BAS. This highlights the importance of closely monitoring kidney function and inflammatory markers in patients receiving this treatment [39]. At the same time, the most common adverse events associated with BAS include gastrointestinal issues, edema, fever, dyspnea, headache, acne, tremor, and insomnia. Additionally, anaphylactic reactions have been reported, suggesting that BAS use should be accompanied by careful patient monitoring [46,47].

In our study, the highest frequencies of ADRs related to drug resistance and transplant rejection in the total cases were registered for BAS, followed by belatacept and THY. A meta-analysis evaluated the efficacy of BAS compared with THY in renal transplantation and found no significant differences in the 1-year acute rejection rates between the two groups; the 1-year graft survival rates were similar. These findings suggest that BAS and THY have comparable efficacy in preventing acute rejection and promoting graft survival in renal transplant patients [48].

Multiple studies confirm our results showing that alemtuzumab has some of the lowest reported rates of resistance, inefficiency, and transplant rejection among total severe ADRs. A study assessing the long-term efficacy and safety of alemtuzumab in treating severe or glucocorticoid-resistant kidney transplant rejection found that over 60% of grafts were successfully preserved with alemtuzumab therapy, demonstrating its effectiveness in managing severe rejection episodes [49]. However, it also reported that graft survival was significantly poorer than that of a reference cohort, and there was a higher risk of serious infections and increased patient mortality associated with alemtuzumab treatment [49]. Another study reviewed kidney transplant rejection rates following a change in immunosuppression regimens due to the COVID-19 pandemic, observing that early follow-up results suggested a possibly higher rejection rate with BAS than with alemtuzumab. However, the authors noted that longer-term follow-up is necessary to draw stronger conclusions [50].

BAS has a higher probability of resistance than other medications, except for belatacept, which did not meet the inclusion criteria for this analysis (Figure 5). A clinical trial evaluating the efficacy of BAS induction therapy in kidney transplant recipients with a low immunological risk profile found that it did not provide a significant benefit in reducing acute rejection rates or improving graft survival within five years post-transplant compared with a non-induction approach [51].

Alemtuzumab and belatacept had a lower probability of reporting drug ineffectiveness than THY and BAS, but no difference was observed between belatacept and alemtuzumab. One study found that alemtuzumab was associated with a lower rate of treatment failure, indicating a lower probability of inefficacy [52]. We assessed other studies based on transplant registries and clinical trials to widen the perspective on the effectiveness of these drugs. We compared the safety signals observed by other clinicians with our results. A long-term (over 20 years) retrospective study on the effectiveness of induction immunosuppression based on the United Network for Organ Sharing Registry Data showed indisputable evidence of the benefits of induction therapy regarding short-term and long-term graft survival. It also captured various induction strategies and regimens recommended through the years. They concluded that depleting antibodies such as alemtuzumab are superior to non-deletional agents (BAS) in significantly reducing the risk of graft failure [53].

Lentile et al. found no significant differences in mortality, graft failure, or all-cause graft survival between participants receiving THY and those receiving BAS over 10 years. To obtain these findings, they employed a data integration approach utilizing a transplant registry database. This method facilitated the comprehensive collection and comparison of long-term outcome data for individuals enrolled in a clinical trial assessing the efficacy of THY versus BAS [54]. A study comparing serious unfavorable outcomes for kidney transplant patients—including death, sepsis, neoplasia, and healthcare assistance—up to a year after undergoing induction immunosuppression showed a favorable profile for THY compared with alemtuzumab and BAS [55]. Another multicenter cohort study on the efficacy and safety of BAS and THY showed similar profiles for both molecules [56].

Approximately 90% of the expert respondents who contributed to the European Consensus on the Management of Sensitized Kidney Transplant Recipients (2024) expressed strong agreement on the clinical value and effectiveness of utilizing alemtuzumab and THY in the treatment of this specific patient population. Their consensus highlights the growing recognition of these therapeutic approaches as essential strategies for improving transplant outcomes, optimizing patient management, and addressing the unique immunological challenges faced by sensitized kidney transplant recipients [57].

Regarding organ rejection, BAS is linked to the highest probability of reporting compared with all other medications (Figure 7). Several studies have shown a significantly higher incidence of acute rejection with BAS than with alemtuzumab [58,59].

According to transplant guidelines, THY is the first recommendation for patients at high risk of rejection, for whom THY seems to be more effective than BAS in preventing the incidence and severity of rejection. However, BAS is an alternative option in different categories: (i) patients unable to tolerate THY (e.g., allergies, leukopenia, thrombocytopenia, or chronic hypotension) [17,60,61]. In patients at low immunologic risk of acute rejection, either BAS or THY represents a reasonable strategy in induction therapy. Research also shows a lower rate of biopsy-proven acute rejection (BPAR) for THY [62] (tacrolimus, mycophenolate, and steroids) [51]. Another explanation for the high rejection rate reported for BAS could be its off-label use. Thus, the EMA and its manufacturer have warned healthcare specialists regarding the off-label use of BAS in cardiac transplants [63]. Moreover, the Summary of Product Characteristics also notes this warning, stating the risk of serious cardiac ADRs such as cardiac arrest and atrial flutter [64].

Furthermore, BAS is used off-label in the prophylaxis of acute rejection in liver or pulmonary transplantation [65,66]. Considering this off-label use, more reports of side effects are expected.

Compared with BAS, a lower probability of reporting transplant rejection was observed for BEL. Researchers have found that its use is associated with higher BPAR rates and grades than cyclosporine. Additionally, other serious adverse events have been observed, including post-transplant lymphoproliferative disorder, progressive multifocal leukoencephalopathy, and tuberculosis. This indicates that while belatacept offers immunosuppressive benefits, it may also introduce significant risks that need to be managed [67]. However, evidence also demonstrates that belatacept is a viable alternative to calcineurin-inhibitor-based immunosuppression [68], and another study suggests belatacept [69]. Moreover, our findings show a higher probability of reporting rejection for belatacept versus other BIIAs (except BAS). Belatacept is currently involved in a pilot, phase I/II clinical trial (ATTAIN) to desensitize kidney transplant candidates with calculated Panel Reactive Antibody (cPRA) values ≥ 99.9%, together with daratumumab, a CD38 monoclonal antibody used in multiple myeloma. Its favorable outcomes have encouraged the continuation of the trial [70].

### Limitations of This Study

Our study is based on data obtained from the EV online platform, and its limitations are common to this kind of approach. An inherent limitation stems from the nature of the spontaneous reporting system, which is prone to under-reporting. This under-reporting can vary based on the specific drug, event type, and severity, ultimately impacting the results. Further skewed reports and lack of data such as age and gender in many ICSRs may also affect our research. Evaluating results in the absence of a clear definition of the population at risk can also lead to an incomplete or distorted process, thus limiting the ability to estimate the true incidence and nature of the observed events. At the same time, our study did not include information on other commonly used drugs; therefore, we could not determine whether the patients used other drugs at the same time. We did not assess a causal relationship between suspected drugs and spontaneously reported adverse reactions, but unlike data obtained from routine clinical trials, all spontaneous reports are suspected cases of adverse reactions. The ability to estimate the actual incidence of adverse events can be affected by biased reporting, often influenced by the phenomenon of under-reporting. Various factors may influence and trigger a failure to report an adverse event during treatment, including lack of awareness of pharmacovigilance, inadequate risk perception, mistrust, guilt, or ignorance. The EMA has observed regional and national differences regarding the variability of spontaneously reported data in EV, which can be attributed to variations in the existence and effectiveness of promotion campaigns [71]. Moreover, certain information regarding clinical characteristics (e.g., BPAR) may be inconsistent or even absent from these reports [72]. Since the EMA acknowledges these limitations, a direct causal correlation cannot be conclusively established based solely on these reports. Moreover, EV explicitly advises against such an approach [73]. Despite all these limitations, our study provides a comprehensive post-marketing analysis of drug safety. The data used were collected from the real world and a large and diverse population through spontaneous reporting systems. The large sample size not only enhances the generalizability of our findings but also helps mitigate potential errors, providing a solid basis for safety assessments. Disproportionality analysis from spontaneous reporting platforms, despite its inherent limitations, is now a validated method used in post-marketing drug safety surveillance.

## 5. Conclusions

Immunosuppressive pharmacotherapy is used both for induction and continuously after transplantation. To ensure accelerated, intense immunosuppression, new biomedicines have been authorized and prescribed for induction, including BAS, THY, alemtuzumab, and belatacept. This study aimed to investigate their ineffectiveness rates, as reported in an international pharmacovigilance database, EV. Ineffectiveness can be reported under various preferred terms accepted by international regulatory authorities.

The number of reported adverse reactions related to ineffectiveness and resistance was low across all four molecules. Compared with all BIIAs, BAS showed high probabilities of drug resistance and transplant rejection being reported. Pharmacovigilance database studies complement clinical trial data and improve the assessment of drug safety profiles. Post-market surveillance data across diverse patient populations and clinical settings offer valuable insights into long-term safety, rare adverse events, and treatment patterns. Thus, pharmacovigilance studies assist clinicians in making decisions based on evidence. Due to the complexity of transplantation procedures and the inherent constraints of observational data, a causal relationship between reported adverse reactions and drug efficacy cannot be implied, and a ranking of these therapeutic agents cannot be established. Further real-world evidence studies will enhance our understanding of the safety and efficacy of these drugs in transplant patients.

## Figures and Tables

**Table 1:** PTs used for reported ADRs related to drug resistance, drug ineffectiveness, or transplant rejection.

ADR	PT
Drug resistance	Drug resistance
	Multiple-drug resistance
Drug ineffectiveness	Drug ineffective
	Drug effect less than expected
	Therapeutic product effect decreased
	Therapeutic product effect incomplete
	Therapeutic product ineffective
	Therapeutic response decreased
	Therapeutic response shortened
	Therapy non-responder
	Therapy partial responder
	Treatment failure
	Decreased activity
Transplant rejection	Bone marrow transplant rejection
	Heart transplant rejection
	Intestine transplant rejection
	Kidney transplant rejection
	Liver transplant rejection
	Lung transplant rejection
	Multiple organ transplant rejection
	Pancreas transplant rejection
	Renal and pancreas transplant rejection
	Skin graft rejection
	Transplant rejection

**Table 2:** Comparison between the general characteristics of reports submitted to the EudraVigilance database. ALE—alemtuzumab; BAS—basiliximab; BEL—belatacept; THY—anti-T lymphocyte immunoglobulin for human use (rabbit); HP—healthcare professional; NS—not specified.

	THY		BAS		ALE		BEL	
	*n*	%	*n*	%	*n*	%	*n*	%
Age								
NS	1085	21.3%	760	21.9%	2858	31.7%	404	41.4%
0–1 Month	7	0.1%	1	0.0%	13	0.1%	2	0.2%
2 Months–2 Years	225	4.4%	81	2.3%	156	1.7%	0	0.0%
3–11 Years	494	9.7%	170	4.9%	209	2.3%	1	0.1%
12–17 Years	335	6.6%	142	4.1%	156	1.7%	3	0.3%
18–64 Years	2590	50.9%	2027	58.4%	5122	56.7%	375	38.4%
65–85 Years	353	6.9%	288	8.3%	504	5.6%	192	19.7%
>85 Years	0	0.0%	0	0.0%	12	0.1%	0	0.00%
Sex								
Female	1900	37.3%	1103	31.8%	5120	56.7%	360	36.9%
Male	2397	47.1%	1910	55.1%	2854	31.6%	500	51.2%
NS	792	15.6%	456	13.1%	1056	11.7%	117	12.0%
Origin								
EEA	1648	32.4%	932	26.9%	5595	62.0%	360	36.9%
NON-EEA	3441	67.6%	2537	73.1%	3435	38.1%	617	63.2%
NS	0	0.0%	0	0.0%	0	0.0%	0	0.0%
Reporter								
HP	4902	96.3%	3342	96.3%	7769	86.0%	893	91.4%
Non-HP	178	3.5%	118	3.4%	1254	13.9%	84	8.6%
Not Specified	9	0.2%	9	0.3%	7	0.1%	0	0.0%

**Table 3:** Comparison of the relative occurrence frequency of each SOC. ALE—alemtuzumab; BAS—basiliximab; BEL—belatacept; THY—anti-T lymphocyte immunoglobulin for human use (rabbit).

System Organ Classes	THY	BAS	ALE	BEL
Blood and lymphatic system disorders	8.6%	5.2%	7.8%	3.7%
Cardiac disorders	3.1%	1.7%	3.4%	2.9%
Congenital, familial, and genetic disorders	0.3%	0.1%	0.2%	0.1%
Ear and labyrinth disorders	0.1%	0.1%	0.4%	0.3%
Endocrine disorders	0.2%	0.1%	4.5%	0.1%
Eye disorders	0.5%	0.5%	1.3%	1.1%
Gastrointestinal disorders	4.3%	4.6%	4.4%	4.0%
General disorders and administration site conditions	11.5%	8.2%	11.9%	8.8%
Hepatobiliary disorders	2.5%	1.7%	1.4%	0.3%
Immune system disorders	10.7%	12.5%	2.9%	8.5%
Infections and infestations	15.4%	20.3%	11.1%	23.1%
Injury, poisoning, and procedural complications	7.3%	7.7%	6.0%	15.0%
Investigations	6.5%	7.6%	8.3%	3.4%
Metabolism and nutrition disorders	1.8%	2.6%	1.4%	1.1%
Musculoskeletal and connective tissue disorders	1.3%	1.1%	3.0%	1.3%
Neoplasms benign, malignant, and unspecified (incl. cysts and polyps)	3.6%	3.7%	2.7%	4.0%
Nervous system disorders	3.3%	2.6%	8.5%	4.4%
Pregnancy, puerperium, and perinatal conditions	0.1%	0.2%	0.3%	0.3%
Product issues	0.1%	0.1%	0.0%	0.5%
Psychiatric disorders	0.6%	0.7%	2.0%	0.8%
Renal and urinary disorders	4.3%	7.47%	2.6%	5.4%
Reproductive system and breast disorders	0.3%	0.3%	0.6%	0.3%
Respiratory, thoracic, and mediastinal disorders	5.6%	4.2%	4.8%	3.7%
Skin and subcutaneous tissue disorders	2.9%	1.9%	7.0%	2.4%
Social circumstances	0.1%	0.0%	0.3%	0.1%
Surgical and medical procedures	0.6%	1.4%	0.6%	2.2%
Vascular disorders	4.7%	3.7%	2.5%	2.5%

## Data Availability

The data are contained in the article.
